# Prior Cancer and Survival in Patients With Esophageal Squamous Cell Carcinoma

**DOI:** 10.1001/jamanetworkopen.2025.60193

**Published:** 2026-02-20

**Authors:** Suna Yu, Ji Taek Hong, Hye-Kyung Jung, Hye Ah Lee, Eui Sun Jeong, Hyuk Lee, Kee Don Choi, Hwoon-Yong Jung, Jun Chul Park, Joong Goo Kwon, Yoon Jin Choi, Su Jin Hong, Jaekyu Sung, Woo Chul Chung, Ki Bae Kim, Seung Young Kim, Kyung Ho Song, Kyung Sik Park, Seong Woo Jeon, Byung-Wook Kim, Han Seung Ryu, Ok-Jae Lee, Gwang Ho Baik, Yong Sung Kim

**Affiliations:** 1 Department of Internal Medicine, Ewha Womans University College of Medicine, Seoul, Korea; 2 Clinical Trial Center, Ewha Womans University Mokdong Hospital, Seoul, Korea; 3 Department of Medicine, Samsung Medical Center, Sungkyunkwan University School of Medicine, Seoul, Korea; 4 Department of Gastroenterology, University of Ulsan College of Medicine, Asan Medical Center, Seoul, Korea; 5 Department of Internal Medicine Institute of Gastroenterology, Yonsei University College of Medicine, Seoul, Korea; 6 Department of Internal Medicine, Daegu Catholic University School of Medicine, Daegu, Korea; 7 Department of Internal Medicine, Seoul National University Bundang Hospital, Gyeonggi-do, Korea; 8 Digestive Disease Center and Research Institute, Soonchunhyang University College of Medicine, Bucheon, Korea; 9 Department of Internal Medicine, Chungnam National University Hospital, Chungnam National University College of Medicine, Daejeon, Korea; 10 Department of Internal Medicine, St. Vincent Hospital, The Catholic University of Korea, Suwon, Korea; 11 Department of Internal Medicine, Chungbuk National University Hospital, Cheongju, Korea; 12 Division of Gastroenterology, Department of Internal Medicine, Korea University Ansan Hospital, Seoul, Korea; 13 Division of Gastroenterology and Hepatology Department of Internal Medicine, Konyang University Hospital, Daejeon, Korea; 14 Department of Internal Medicine, Keimyung University College of Medicine, Daegu, Korea; 15 Department of Internal Medicine, School of Medicine, Kyungpook National University, Daegu, Korea; 16 Department of Internal Medicine, Incheon St. Mary’s Hospital, College of Medicine, The Catholic University of Korea, Incheon, Korea; 17 Department of Internal Medicine and Digestive Disease Research Institute, Wonkwang University School of Medicine, Iksan, Korea; 18 Department of Internal Medicine, Gyeongsang National University College of Medicine and Institute of Medical Sciences, Gyeongsang National University, Jinju, Korea; 19 Department of Internal Medicine, Chuncheon Sacred Heart Hospital, Hallym University College of Medicine, Chuncheon, Korea; 20 Wonkwang Digestive Disease Research Institute, Gunpo, Korea

## Abstract

**Importance:**

Esophageal squamous cell carcinoma (ESCC) is highly prevalent in Asian populations and carries a poor prognosis. With growing numbers of cancer survivors, the prognostic impact of prior cancer in ESCC remains unclear. Most existing data are derived from Western cohorts dominated by adenocarcinoma, limiting generalizability to Asian populations.

**Objective:**

To evaluate whether prior cancer is associated with overall survival (OS) and esophageal cancer–specific mortality (ECSM) in a nationwide Korean ESCC cohort.

**Design, Setting, and Participants:**

A retrospective cohort study of patients with newly diagnosed ESCC across 19 tertiary hospitals in Korea from 2005 to 2017 was conducted. Follow-up was completed in 2017. Data were reanalyzed in August 2025. Exclusion criteria were nonsquamous histology (including adenocarcinoma), diagnosis of esophageal cancer within 6 months of a prior cancer, multiple prior cancers, and hematologic cancers.

**Exposures:**

History of cancer before the diagnosis of ESCC, classified by cancer type and latency (≤5 years vs >5 years).

**Main Outcomes and Measures:**

The primary outcome was OS, and the secondary outcome was esophageal cancer–specific mortality (ECSM). Hazard ratios (HRs) and cause-specific hazard ratios (CSHRs) were estimated after adjustment for clinicopathologic and treatment variables. Propensity score–adjusted Cox regression and competing risk regression models were used. Subgroup analyses were conducted by prior cancer type and latency period.

**Results:**

Of the 5557 patients (mean [SD] age, 64.7 [8.9] years; 5168 [93.0%] male), 368 (6.6%) had a prior cancer and were older and more often diagnosed at an earlier stage than those without prior cancer. Patients with a prior cancer had significantly poorer outcomes, with a median OS of 3.58 (95% CI, 2.50–4.92) vs 4.25 (95% CI, 3.83–4.58) years and a 3-year ECSM of 8.35% (95% CI, 4.42%-12.29%) vs 4.98% (95% CI, 4.17%-5.78%) compared with those without a prior cancer. Prior cancer was independently associated with worse OS (HR, 1.25; 95% CI, 1.07–1.47) and ECSM (CSHR, 1.89; 95% CI, 1.09–3.29). Among prior cancer types, patients with a history of stomach, head and neck, or lung cancer demonstrated poorer OS (HR, 1.63; 95% CI, 1.24-2.15; *P* < .001). A latency of 5 or more years was also associated with reduced OS (HR, 1.27; 95% CI, 1.03–1.57; *P* = .02).

**Conclusions and Relevance:**

In this nationwide Korean cohort study, prior cancer was an independent adverse prognostic factor in ESCC, with stomach, head and neck, and lung cancers associated with the poorest outcomes.

## Key Points

**Question:**

Is a history of cancer independently associated with survival outcomes in patients with esophageal squamous cell carcinoma (ESCC)?

**Findings:**

In this cohort study of 5557 patients with newly diagnosed ESCC, 6.6% had a prior cancer. Prior cancer was independently associated with poorer overall and cancer-specific survival and with prior stomach, head and neck, or lung cancers; the negative association with survival was also found when the prior cancer occurred more than 5 years before ESCC diagnosis.

**Meaning:**

In this study, prior cancer was associated with worse prognosis in ESCC, suggesting the need for careful clinical consideration in cancer survivors who develop ESCC.

## Teaser

This cohort study evaluates the association between prior cancer diagnosis and survival among patients with esophageal squamous cell carcinoma.

## Introduction

Second primary cancer (SPC), defined as a new cancer arising in a different organ or tissue at least 6 months after the initial cancer, is increasingly recognized as a challenge in oncology.^1,2^ The number of cancer survivors continues to increase due to earlier detection and advances in treatment, resulting in a growing population at risk for SPCs.^1^ Esophageal cancer is among the most lethal cancers worldwide, ranking seventh in incidence and sixth in cancer-related mortality,^3,4^ with 5-year survival rates rarely exceeding 30%.^5,6,7,8^

Although prior cancer has been studied in several cancers, evidence regarding its prognostic impact in esophageal cancer remains limited and inconsistent.^9,10^ A population-based Surveillance, Epidemiology, and End Results analysis reported comparable survival between patients with and without prior cancer, with most deaths attributable to esophageal cancer itself rather than the antecedent tumor.^9^ However, that study was predominantly based on Western cohorts with adenocarcinoma histology, which differs markedly from squamous cell carcinoma (SCC) in epidemiology, risk factors, and biology.^3,11^ In Asia, where SCC predominates, data on the prognostic implications of prior cancer in esophageal cancer are scarce.^3^

Given these gaps, clarifying the prognostic impact of prior cancer in Asian individuals with esophageal squamous cell carcinoma (ESCC) is clinically important. Moreover, understanding how outcomes vary according to the type and latency of prior cancer may provide evidence to optimize surveillance strategies and improve disease management in patients.^1,2^

Therefore, this nationwide multicenter study aimed to evaluate the association of prior cancer with overall survival (OS) and esophageal cancer–specific mortality (ECSM) in patients with ESCC, and to investigate the differential effects according to type of prior cancer and latency period. We hypothesized that patients with a prior cancer would have worse survival outcomes than those without such history.

## Methods

### Study Design and Setting

This retrospective multicenter cohort study included patients newly diagnosed with esophageal cancer between January 1, 2005, and December 31, 2017. Follow-up was completed in 2017, and statistical analyses were repeated in 2025. The study was approved by the Institutional Review Boards of all participating centers. The need for informed consent was waived because data were anonymized before analysis. This study was conducted in accordance with the principles of the Declaration of Helsinki^12^ and the International Conference on Harmonization Guidelines for Good Clinical Practice guidelines.^13^ This report follows the Strengthening the Reporting of Observational Studies in Epidemiology (STROBE) reporting guideline for cohort studies.

Nineteen tertiary referral hospitals across Korea were selected using a cluster sampling strategy to ensure regional representation. In Korea, the care of patients with ESCC is largely centralized within tertiary referral hospitals, which is relevant for interpreting nationwide patterns of diagnosis and outcomes. Patients were identified from institutional databases using *International Statistical Classification of Diseases and Related Health Problems, Tenth Revision (ICD-10)* codes C15.0–C15.9.^14^

**Figure 1.**
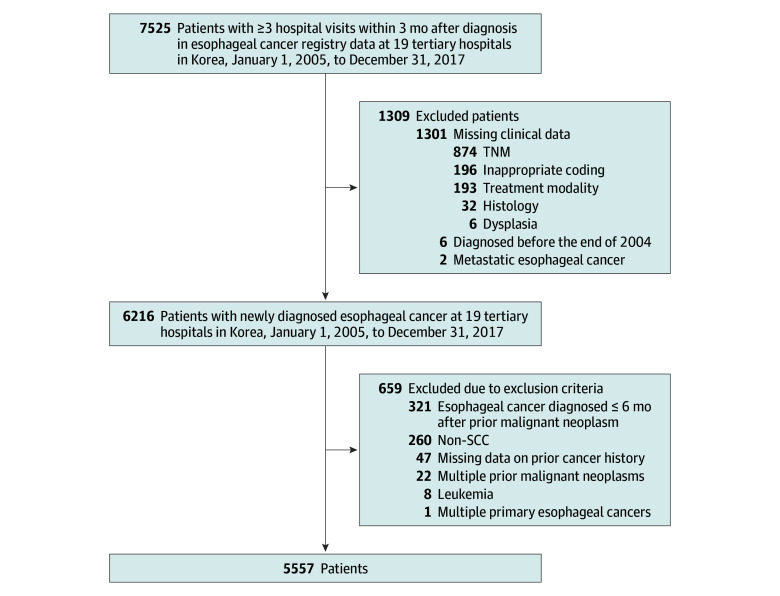
Study Flow Diagram Non-SCC indicates nonsquamous cell carcinoma; TNM, tumor node metastasis.

Inclusion criteria were pathologically confirmed ESCC and at least 3 hospital visits within 3 months after diagnosis to enhance diagnostic specificity. Exclusion criteria were nonsquamous histology (including adenocarcinoma), diagnosis of esophageal cancer within 6 months of a prior cancer, multiple prior cancers, and hematologic cancers. The study flow diagram appears as Figure 1.

### Data Collection

Clinical data were deidentified and collected using a standardized case report form. Variables included age, sex, smoking status,^15,16^ comorbidities (such as liver cirrhosis and cerebrovascular disease), tumor histology and grade, anatomic location, clinical stage, treatment modality, and follow-up outcomes. Tumor location was categorized as upper, middle, or lower third of the esophagus. Clinical stage was determined according to the 7th edition of the American Joint Committee on Cancer tumor node–metastasis staging system, based on endoscopy, endoscopic ultrasonography, computed tomography, and positron emission tomography findings.^17^

Treatment modalities included endoscopic resection, surgery, surgery with perioperative therapy, definitive concurrent chemoradiotherapy (CCRT), and best supportive care. Endoscopic resection included mucosal or submucosal dissection. Surgical procedures were performed according to standard techniques, including Ivor Lewis, McKeown, or trans-hiatal esophagectomy.

### Outcomes

The primary outcome was OS, defined as the interval from diagnosis to death from any cause. The secondary outcome was ECSM. Vital status and cause of death were ascertained through hospital records and linkage to the Korean National Statistical Office death registry.

### Statistical Analysis

Patients were categorized according to cancer history. Baseline characteristics were compared using χ^2^ and *t* tests. OS according to cancer history was assessed using the Kaplan-Meier method with log-rank testing, and ECSM was evaluated using cause-specific Cox regression. Cox proportional hazards regression was used to identify prognostic factors. Sex, age, smoking status, liver cirrhosis, cerebrovascular disease, tumor grade, tumor location, and clinical stage were ultimately included in the multivariate analysis. The proportional hazard assumption of the Cox proportional hazard models was evaluated using log–minus–log survival plots and Schoenfeld residuals. Cause-specific hazard ratios (CSHRs) for ECSM were estimated using Cox proportional hazard models, treating deaths from other causes as competing events. Propensity score matching was performed to minimize confounding, using sex, age, smoking, liver cirrhosis, cerebrovascular disease, tumor grade, location, clinical stage, and treatment modality.^18^ Subgroup analyses were performed to evaluate OS according to prior cancer type (stomach; head and neck/lung; colorectal/breast/prostate; others) and latency period. Additionally, the 2-way interaction between prior cancer type and latency period was assessed. All tests were 2-sided, with *P* < .05 considered statistically significant. Analyses were performed using R version 4.5.1 software (R Foundation for Statistical Computing).

## Results

### Baseline Characteristics

A total of 5557 patients with ESCC were identified (mean [SD] age, 64.7 [8.9] years; 5168 [93.0%] male; 389 [7.0%] female), of whom 368 (6.6%) had a prior cancer (Table 1). Patients with prior cancer were older (mean [SD] age, 67.3 [8.8] years) compared with patients without prior cancer (mean [SD] age, 64.6 [8.9] years). Tumor location differed between groups: the lower third predominated in patients without a prior cancer (2252 of 5189 [44.3%]), whereas the middle third was most common among those with a prior cancer (151 of 368 [41.3%]). Moderately differentiated (grade 2) tumors were most frequent in both groups. Stage I disease was more common in patients with a prior cancer (143 of 368 [38.9%] vs 1610 of 5189 [31.0%]; *P* = .02) whereas stages II through IV were more frequent in those without. Endoscopic resection was more often performed in the prior cancer group (15 of 368 [14.7%] vs 254 of 5189 [4.9%]; *P* < .001), whereas surgery alone (77 of 368 [20.9%] vs 1671 of 5189 [32.2%]; *P* < .001) and CCRT (136 of 368 [37.0%] vs 1290 of 5189 [24.9%]; *P* < .001) were less common. The most frequent prior cancers were stomach (118 of 368 [32.1%]), head and neck (71 of 368 [19.3%]), colorectal (46 of 368 [12.5%]), lung (29 of 368 [7.9%]), and liver (28 of 368 [7.6%]) (eTable 1 in Supplement 1).

**Table 1.** Baseline Characteristics of 5557 Patients With Esophageal Squamous Cell Carcinoma

Characteristic	Prior cancer, No. (%)		*P* value
	No (n = 5189 [93.4%])	Yes (n = 368 [6.6%])	
Age, mean (SD), y	64.6 (8.9)	67.3 (8.8)	<.001
Sex			
Male	4834 (93.2)	334 (90.8)	.08
Female	355 (6.8)	34 (9.2)	
Smoking status			
Current	1457 (29.0)	88 (24.9)	.08
Never	1026 (20.4)	101 (28.6)	
Former	2547 (50.6)	164 (46.5)	
Comorbidity			
Chronic kidney failure	40 (0.8)	3 (0.8)	.76
Liver cirrhosis	142 (2.7)	24 (6.5)	<.001
Ischemic heart disease	128 (2.5)	20 (5.4)	.001
Cerebrovascular disease	158 (3.0)	9 (2.4)	.51
SEER histologic grade			
G1, well differentiated	700 (14.9)	63 (19.3)	.03
G2, moderately differentiated	3379 (71.9)	213 (65.3)	
G3, poorly differentiated	621 (13.2)	50 (15.3)	
T stage			
T1	1795 (35.0)	154 (42.5)	.02
T2	1169 (22.8)	73 (20.2)	
T3	1861 (36.3)	110 (30.4)	
T4	300 (5.9)	25 (6.9)	
N stage			
N0	2409 (46.9)	209 (57.7)	.001
N1	2200 (42.8)	127 (35.1)	
N2	381 (7.4)	19 (5.3)	
N3	145 (2.8)	7 (1.9)	
M stage			
M0	4617 (89.0)	330 (89.7)	.68
M1	572 (11.0)	38 (10.3)	
*AJCC 7* clinical stage			
I	1610 (31.0)	143 (38.9)	.02
II	1567 (30.2)	100 (27.2)	
III	1440 (27.8)	87 (23.6)	
IV	572 (11.0)	38 (10.3)	
Clinical stage			
IA	308 (5.9)	42 (11.4)	.003
IB	1302 (25.1)	101 (27.4)	
IIA	392 (7.6)	30 (8.2)	
IIB	1175 (22.6)	70 (19.0)	
IIIA	946 (18.2)	57 (15.5)	
IIIB	212 (4.1)	12 (3.3)	
IIIC	282 (5.4)	18 (4.9)	
IV	572 (11.0)	38 (10.3)	
Tumor location			
Upper	851 (16.8)	75 (20.6)	.05
Middle	1927 (37.9)	151 (41.4)	
Lower	2252 (44.3)	136 (37.3)	
Esophagogastric junction	49 (1.0)	3 (0.8)	
Treatment method			
Endoscopic resection	254 (4.9)	54 (14.7)	<.001
Surgery alone	1671 (32.2)	77 (20.9)	
Surgery and chemotherapy	1229 (23.8)	43 (11.7)	
Definitive CCRT	1290 (24.9)	136 (37.0)	
Others	117 (2.3)	11 (3.0)	
Best supportive care	628 (12.1)	47 (12.8)	

Abbreviations: AJCC, American Joint Committee on Cancer; *AJCC 7, AJCC Cancer Staging Manual*, 7th edition; CCRT, concurrent chemoradiation therapy: SEER, Surveillance, Epidemiology, and End Results.

### All-Cause and Esophageal Cancer-Specific Survival

**Figure 2.**
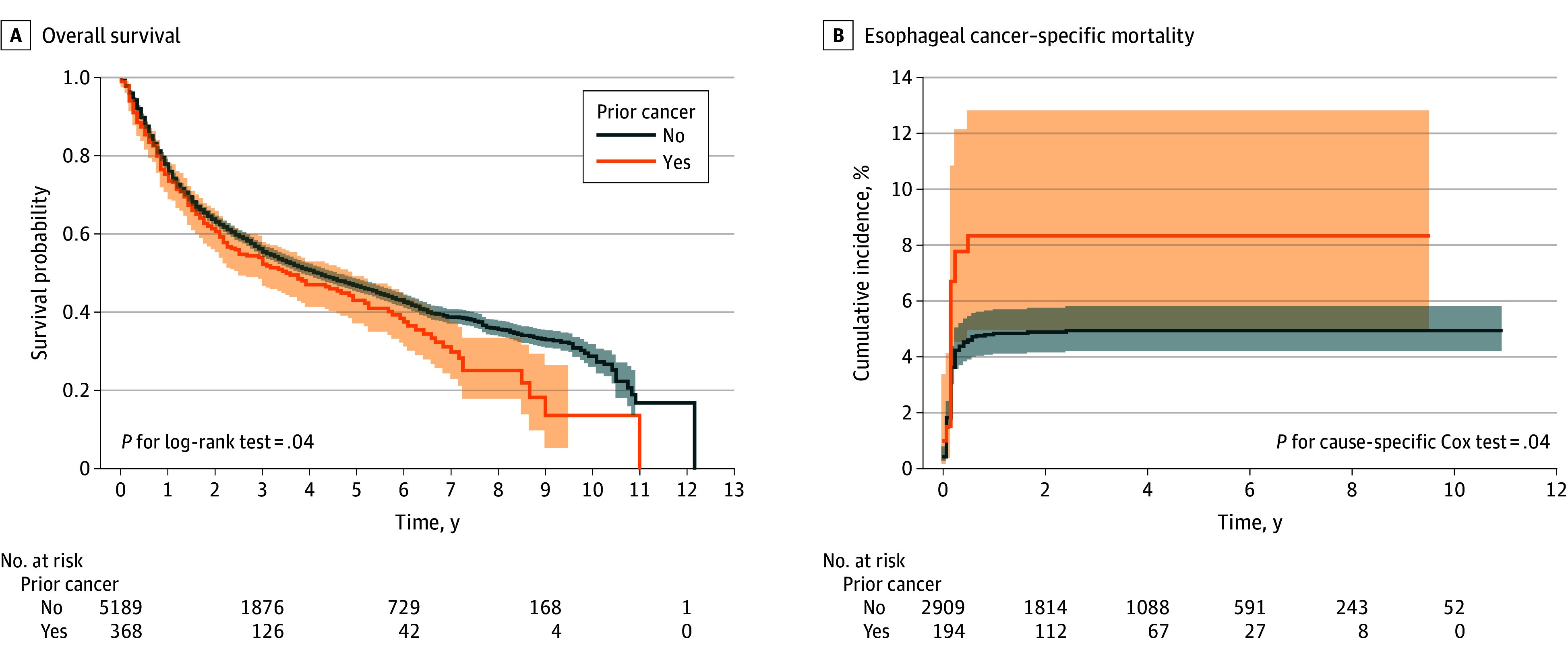
Survival Probability and Cumulative Incidence of Esophageal Cancer-Specific Mortality

Patients with a prior cancer had poorer OS and ECSM than those without a prior cancer (Figure 2). The median OS was 4.25 (95% CI, 3.83–4.58) years in patients without a prior cancer and 3.58 (95% CI, 2.50–4.92) years in those with a prior cancer (log-rank *P* = .04). For esophageal cancer–specific mortality, the 3-year cumulative incidence was 4.98% (95% CI, 4.17%-5.78%) in the group without a prior cancer and 8.35% (95% CI, 4.42%-12.29%) in the group with a prior cancer.

### Association of Prior Cancer With OS After Multivariable Adjustment

In a multivariable Cox regression model adjusting for sex, age, smoking status, liver cirrhosis, cerebrovascular disease, histologic grade, clinical stage, and tumor location, prior cancer was independently associated with worse OS (hazard ratio [HR], 1.25; 95% CI, 1.07-1.47; *P* = .006) and ECSM (CSHR, 1.89; 95% CI, 1.09-3.29; *P* = .02) (Table 2; eTable 2 in Supplement 1).

**Table 2.** Multivariable Covariate-Adjusted Cox Models for Overall Survival and Esophageal Cancer-Specific Mortality for Esophageal Squamous Cell Carcinoma^a^

Characteristic	Overall survival		Esophageal cancer-specific survival	
	Multivariate, HR (95% CI)	*P* value	Multivariate, cause-specific HR (95% CI)	*P* value
Prior cancer				
No	1 [Reference]		1 [Reference]	NA
Yes	1.25 (1.07-1.47)	.006	1.89 (1.09-3.29)	.02
Age, mean (SD)	1.03 (1.02-1.03)	<.001	1.03 (1.00-1.05)	.02
Sex				
Male	0.98 (0.82-1.18)	.86	0.88 (0.44-1.77)	.73
Female	1 [Reference]		1 [Reference]	NA
Smoking status				
Current	1.22 (1.08-1.37)	.001	1.33 (0.81-2.18)	.27
Never	1 [Reference]		1 [Reference]	NA
Former	1.00 (0.89-1.12)	.97	0.74 (0.46-1.18)	.20
Comorbidity				
Liver cirrhosis	1.41 (1.15-1.73)	.001	1.88 (0.88-4.00)	.10
Cerebrovascular disease	1.31 (1.06-1.63)	.01	0.81 (0.32-2.00)	.64
SEER histologic grade				
G1, well differentiated	1 [Reference]		1 [Reference]	NA
G2, moderately differentiated	1.08 (0.95-1.22)	.24	1.16 (0.66-2.04)	.61
G3, poorly differentiated	0.98 (0.84-1.14)	.78	1.12 (0.58-2.14)	.74
*AJCC 7* clinical stage				
I	1 [Reference]		1 [Reference]	NA
II	2.62 (2.29-2.98)	<.001	2.77 (1.43-5.34)	.002
III	4.58 (4.02-5.21)	<.001	9.83 (5.46-17.70)	<.001
IV	8.49 (7.29-9.89)	<.001	33.56 (17.80-63.26)	<.001
Tumor location				
Upper	1 [Reference]		1 [Reference]	NA
Middle	1.04 (0.92-1.17)	.56	1.96 (1.18-3.23)	.009
Lower	0.93 (0.83-1.05)	.25	1.08 (0.64-1.82)	.78
Esophagogastric junction	0.81 (0.52-1.26)	.35	0.98 (0.13-7.35)	.98

Abbreviations: AJCC, American Joint Committee on Cancer; *AJCC 7, AJCC Cancer Staging Manual*, 7th edition; HR, hazard ratio; NA, not applicable; SEER, Surveillance, Epidemiology, and End Results.

^a^ Adjusted for sex, age, smoking, liver cirrhosis, cerebrovascular disease, grade (differentiation), clinical stage, and tumor location.

### Association After Propensity Score Matching

Following propensity score matching (n = 628), prior cancer was associated with worse OS (HR, 1.30; 95% CI, 1.04-1.64; *P* = .02) and ECSM (CSHR, 7.10; 95% CI, 1.97-25.65; *P* = .003) (eTable 3 in Supplement 1).

### Findings Based on the Type and Latency of Prior Cancer

Survival outcomes varied depending on the type of prior cancer. In stomach cancer, patients were older (mean [SD] age, 69.21 [8.1] years vs 64.57 [8.9] years) and more frequently had cirrhosis (7 of 118 [5.9%] vs 142 of 5189 [2.7%]). Stage I and endoscopic resection rates were higher (57 of 118 [48.3%] vs 1610 of 5189 [31%] and 28 of 118 [23.7%] vs 254 of 5189 [4.9%]), but overall surgery rates were lower (24 of 118 [ 20.3%] vs 1671 of 5189 [4.9%]). Prior stomach cancer was associated with poorer ECSM (CSHR, 2.63; 95% CI; 1.27-5.44; *P* = .009) (eTables 4 and 5 in Supplement 1). In lung cancer and head and neck cancer, stage IV disease and CCRT use were more common (16 of 100 [16%] vs 572 of 5189 [11%] and 50 of 100 [50.0%] vs 1290 of 5189 [24.9%]) (eTable 4 in Supplement 1). These cancers were associated with worse OS (HR, 1.63; 95% CI, 1.24-2.15; *P* < .001) (eTable 6 in Supplement 1). In colorectal, breast, prostate, and other cancers, no significant differences were observed for OS or ECSM.

**Figure 3.**
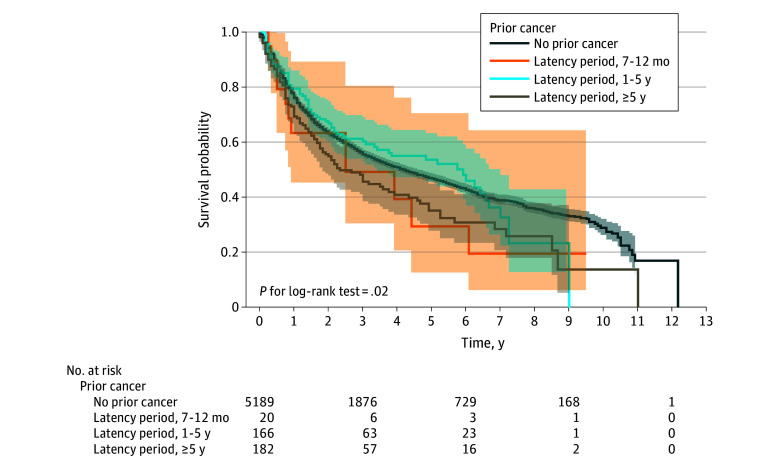
Diagnostic Time and Prior Cancer in Overall Survival

For the latency period, latency analysis showed that patients who developed 5 or more years after their prior cancer had significantly worse OS compared with those without prior cancer (Bonferroni adjusted log-rank *P* = .02), whereas shorter latency periods were not associated with a significant difference (Figure 3). An independent association was found in the multivariable Cox regression model (HR, 1.27; 95% CI, 1.03–1.57; *P* = .02). With regard to interaction, there was no significant interaction between prior cancer type and latency period in relation to OS (*P* = .31 for interaction).

## Discussion

In this large nationwide cohort study, we found that a history of cancer was associated with the poorer prognosis of patients with ESCC. Both OS and ECSM were significantly worse among patients with prior cancers, and these findings remained robust after multivariable adjustment and propensity score–matched analyses. Notably, prior stomach, head and neck, and lung cancers were associated with poorer outcomes. Importantly, ESCC diagnosed more than 5 years after stomach cancer was associated with worse prognosis, suggesting that conventional surveillance periods may be insufficient.

Our results align with several prior studies^19,20,21,22,23^ demonstrating poorer cancer-specific survival in patients with antecedent cancers such as lung, nasopharyngeal, pancreatic, and breast cancers. Conversely, other reports have suggested similar or even better outcomes in survivors with subsequent ESCC. For example, Pan et al^9^ observed that patients with prior cancers had improved cancer-specific survival compared with those with primary ESCC, likely reflecting enhanced surveillance and health awareness among cancer survivors. Wang et al^24^ reported improved cancer-specific survival among patients with nasopharyngeal carcinoma and antecedent cancers. Such discrepancies may stem from differences in tumor biology, cancer type, and regional epidemiology. Aggressive prior tumors (eg, lung, pancreatic) may negatively impact outcomes, whereas indolent tumors (eg, prostate, thyroid) may exert little effect.

Our study helps clarify these inconsistencies by focusing exclusively on ESCC in an Asian population, where the distribution of prior cancers differs significantly from that in the Western populations. While prostate and bladder cancers predominate in Western cohorts, stomach cancer was the most frequent antecedent cancer in our population, followed by head and neck and lung cancers. This difference may account for the divergent associations observed across regions.

The association of prior stomach cancer with worse survival may be explained by several mechanisms. First, long-term mucosal changes following gastrectomy—including bile reflux, altered gastric microbiota, and chronic inflammation—can create a procarcinogenic environment in the esophagus. Molecular alterations identified in large-scale genomic characterizations,^25^ such as p53 mutations and abnormal DNA methylation patterns, have been reported in metachronous esophageal tumors after gastrectomy.^26,27,28,29,30,31,32^ These biological pathways may partly explain why the risk of ESCC persists beyond the conventional 5-year surveillance window.

The association of ESCC with head and neck or lung cancers supports the concept of field cancerization in the aerodigestive tract.^33^ Shared exposures, such as alcohol, tobacco, and environmental carcinogens, induce widespread epithelial damage, predisposing individuals to synchronous and metachronous tumors in anatomically contiguous regions.^34,35,36,37,38,39^ Prior studies have found that head and neck cancer survivors have up to an 8-fold increased risk of developing ESCC,^40^ and image-enhanced endoscopy has revealed high rates of metachronous esophageal lesions in this population.^41,42^ These findings reinforce the biological plausibility of our results.

Our findings have several important clinical implications. For stomach cancer survivors, the conventional 5-year endoscopic surveillance recommended by Japanese guidelines^43^ may be insufficient. Data suggest that the risk of second primary esophageal cancer persists for up to 20 years,^44,45,46^ highlighting the need for extended surveillance protocols. In clinical practice, this may translate into stratified follow-up strategies, with long-term endoscopic surveillance prioritized for high-risk subgroups, such as older males, smokers, or those with prior T4 gastric cancer.

For patients with a history of head and neck or lung cancers, our data suggest that systematic screening for ESCC should be considered. Since many of these patients present with advanced ESCC and often undergo definitive CCRT rather than surgery, earlier detection could substantially improve treatment options and outcomes. Incorporating esophageal screening into survivorship care plans—using image-enhanced endoscopy—may facilitate earlier diagnosis of asymptomatic lesions and improve survival.^47,48,49,50^

At the public health level, the increasing number of cancer survivors in aging populations highlights the importance of addressing SPCs. Our data suggest that ESCC as an SPC is not a random occurrence but follows a pattern based on the type of prior cancer and latency.^51^ Surveillance guidelines, which currently focus on recurrence of the index cancer, should be updated to incorporate the risk of SPCs, particularly in high-risk organs such as those in the aerodigestive tract.

### Strengths and Limitations

The strengths of this study include its large sample size, nationwide multicenter design, and exclusive focus on ESCC, thereby avoiding histological heterogeneity associated with adenocarcinoma. Furthermore, we adjusted for key lifestyle risk factors, such as smoking and alcohol consumption, which are frequently missing in registry-based studies but critically relevant for ESCC.^52^

Several limitations must be acknowledged. First, the retrospective design introduces a risk of selection bias. Second, detailed clinical characteristics of prior cancers, such as treatment type, stage, and response, were unavailable, which may influence survival outcomes. Third, these findings should be interpreted within the context of the Korean health care system, where the management of ESCC is highly centralized to tertiary referral hospitals, potentially limiting generalizability and influencing diagnostic pathways, treatment decisions, and clinical outcomes

## Conclusions

In this nationwide Korean cohort study, a history of cancer was independently associated with worse prognosis in patients with ESCC. In particular, poorer OS was found in patients with a history of stomach, head and neck, or lung cancer. These findings highlight the prognostic relevance of prior cancer and suggest that further research is warranted to clarify the underlying mechanisms and potential clinical implications.

## Notes

Supplement 1. **eTable 1.** Distribution of prior malignancy types **eTable 2.** Univariate analysis of the impact of baseline risk factors, including prior malignancy history, on overall survival **eTable 3.** Baseline characteristics after propensity score matching **eTable 4.** Baseline characteristics of patients with esophageal squamous cell carcinoma according to the type of prior malignancy **eTable 5.** Impact of prior stomach cancer on the prognosis of subsequent primary esophageal squamous cell carcinoma **eTable 6.** Impact of prior lung cancer and head & neck cancer on the prognosis of subsequent primary esophageal squamous cell carcinoma

Supplement 2.
Data Sharing Statement
