# Supplementary material for: Prior Cancer and Survival in Patients With Esophageal Squamous Cell Carcinoma
Source: JAMA Netw Open. 2026 Feb 20;9(2):e2560193. doi: 10.1001/jamanetworkopen.2025.60193 (PMC12924099; doi:10.1001/jamanetworkopen.2025.60193)
Supplement: Supplement 1. — eTable 1. Distribution of prior malignancy types eTable 2. Univariate analysis of the impact of baseline risk factors, including prior malignancy history, on overall survival eTable 3. Baseline characteristics after propensity score matching eTable 4. Baseline characteristics of patients with esophageal squamous cell carcinoma according to the type of prior malignancy eTable 5. Impact of prior stomach cancer on the prognosis of subsequent primary esophageal squamous cell carcinoma eTable 6. Impact of prior lung cancer and head & neck cancer on the prognosis of subsequent primary esophageal squamous cell carcinoma [file jamanetwopen-e2560193-s001.pdf]

Supplemental Online Content

Yu S, Hong JT, Jung H, et al. Prior cancer and survival in patients with esophageal squamous cell carcinoma. *JAMA Netw Open.* 2026;9(2):e2560193. doi:10.1001/jamanetworkopen.2025.60193

eTable 1. Distribution of Prior Malignancy Types

eTable 2. Univariate analysis of the impact of baseline risk factors, including prior malignancy history, on overall survival

eTable 3. Baseline characteristics After Propensity Score Matching

eTable 4. Baseline characteristics of patients with esophageal squamous cell carcinomar according to the type of prior malignancy

eTable 5. Impact of prior stomach cancer on the prognosis of subsequent primary esophageal squamous cell carcinoma

eTable 6. Impact of prior lung cancer and head & neck cancer on the prognosis of subsequent primary esophageal squamous cell carcinoma

This supplemental material has been provided by the authors to give readers additional information about their work.

© 2026 Yu S et al. *JAMA Network Open*.

eTable 1. Distribution of Prior Malignancy Types

	N = 368	%
Stomach cancer	118	32.1
Head & Neck cancer	71	19.3
Colorectal cancer	46	12.5
Lung cancer	29	7.9
Liver cancer	28	7.6
Prostate cancer	18	4.9
Breast cancer	17	4.6
Bladder ureter cancer	16	4.4
Thyroid cancer	11	3.0
Other cancer	3	0.8
Pancreatic cancer	3	0.8
Brain cancer	2	0.4
Renal cancer	2	0.5
Skin cancer	2	0.54
Cervix cancer	1	0.3
GB biliary cancer	1	0.3

© 2026 Yu S et al. *JAMA Network Open*.

eTable 2. Univariate analysis of the impact of baseline risk factors, including prior malignancy history, on overall survival

	Univariate			
Characteristics	HR	low 95% CI	upper 95% CI	p-value
Prior malignancy				
No	ref			
Yes	1.16	1.00	1.34	0.05
Age (mean, SD)	1.03	1.02	1.03	<.001
Sex				
Male	1.11	0.95	1.30	0.21
Female	ref			
Smoking				
Current smoker	1.21	1.09	1.34	<.001
Non-smoker	ref			
Ex-smoker	0.93	0.84	1.03	0.14
Comorbidity				
Chronic renal failure	1.10	0.72	1.67	0.66
Liver cirrhosis	1.45	1.20	1.75	<.001
Ischemic heart disease	1.14	0.92	1.41	0.24
Cerebrovascular disease	1.40	1.15	1.71	<.001
Grade (differentiation)				
G1, well	ref			
G2, moderately	1.31	1.16	1.47	<.001
G3, poorly	1.65	1.42	1.91	<.001
T stage				
T1	ref			
T2	2.38	2.11	2.68	<.001
T3	4.28	3.85	4.76	<.001
T4	5.05	4.31	5.92	<.001
N stage				
N0	ref			
N1	2.40	2.20	2.61	<.001
N2	2.98	2.58	3.44	<.001
N3	3.80	3.08	4.69	<.001
M stage				
M0	ref			
M1	3.33	3.01	3.67	<.001
Clinical stage				

© 2026 Yu S et al. *JAMA Network Open*.

stage I	ref			
stage II	2.53	2.24	2.86	<.001
stage III	4.39	3.90	4.94	<.001
stage IV	7.90	6.91	9.03	<.001
Tumor location				
Upper	ref			
Middle	0.92	0.83	1.03	0.14
Lower	0.78	0.70	0.86	<.001
EG junction	0.69	0.46	1.04	0.08
Treatment Method				
Operation	ref			
Endoscopic resection	0.53	0.39	0.73	<.001
Adjuvant	2.06	1.77	2.39	<.001
Neoadjuvant	2.32	2.01	2.67	<.001
CCRT	4.13	3.69	4.62	<.001
Others	2.35	1.80	3.07	<.001
Best supportive care	6.13	5.39	6.98	<.001

CCRT, concurrent chemoradiation therapy; CI, confidence interval; HR, hazard ratio; SD, standard deviation

© 2026 Yu S et al. *JAMA Network Open*.

eTable 3. Baseline characteristics After Propensity Score Matching

	Prior malignancy				
	No (n=314, 50.0%)		Yes (n=314, 50.0 %)		
Characteristics	n	%	n	%	p-value
Age (mean, SD)	67.61	8.0	67.23	8.6	0.57
Sex					
Male	276	87.9	282	89.8	0.45
Female	38	12.1	32	10.2	
Smoking					
Current smoker	72	22.9	79	25.2	0.73
Non-smoker	91	29.0	93	29.6	
Ex-smoker	151	48.1	142	45.2	
Comorbidity					
Liver cirrhosis	17	5.4	20	6.4	0.61
Cerebrovascular disease	3	1.0	8	2.6	0.13
Grade (differentiation)					
G1, well	62	19.8	59	18.8	0.95
G2, moderately	205	65.3	208	66.2	
G3, poorly	47	15.0	47	15.0	
Clinical stage					
stage I	120	38.2	116	36.9	0.63
stage II	93	29.6	90	28.7	
stage III	80	25.5	78	24.8	
stage IV	21	6.7	30	9.6	
Tumor location					
Upper	51	16.2	61	19.4	0.41
Middle	126	40.1	130	41.4	
Lower	136	43.3	120	38.2	
EG junction	1	0.3	3	1.0	
Treatment Method					
Operation	78	24.8	71	22.6	0.99
Endoscopic resection	42	13.4	41	13.1	
Adjuvant	17	5.4	19	6.1	
Neoadjuvant	20	6.4	17	5.4	
CCRT	113	36.0	119	37.9	
Others	6	1.9	7	2.2	
Best supportive care	38	12.1	40	12.7	

CCRT, concurrent chemoradiation therapy; SD, standard deviation

© 2026 Yu S et al. *JAMA Network Open*.

eTable 4. Baseline characteristics of patients with esophageal squamous cell carcinomar according to the type of prior malignancy

Prior malignancy	No (n=5189, 93.4%)		Stomach cancer (n=118, 2.1%)		lung & HN cancer (n=100, 1.8%)		Colorectal & Breast & Prostate (n=81, 1.5%)		Other cancer (n=69, 1.2%)		
Characteristics	n	%	n	%	n	%	n	%	n	%	p-value
Age (mean, SD)	64.57	8.9	69.21	8.1	64.80	9.5	68.00	7.5	66.67	9.6	<.001
Sex											
Male	4834	93.2	115	97.5	97	97.0	60	74.1	62	89.9	<.001
Female	355	6.8	3	2.5	3	3.0	21	25.9	7	10.1	
Smoking											
Current smoker	1457	29.0	34	30.4	22	22.7	15	19.2	17	25.8	0.001
Non-smoker	1026	20.4	25	22.3	25	25.8	32	41.0	19	28.8	
Ex-smoker	2547	50.6	53	47.3	50	51.5	31	39.7	30	45.5	
Comorbidity											
Chronic renal failure	40	0.8	0	0.0	0	0.0	0	0.0	3	4.3	0.08
Liver cirrhosis	142	2.7	7	5.9	3	3.0	4	4.9	10	14.5	<.001
Ischemic heart disease	128	2.5	3	2.5	3	3.0	10	12.3	4	5.8	<.001
Cerebrovascular disease	158	3.0	3	2.5	3	3.0	3	3.7	0	0.0	0.69
Grade (differentiation)											
G1, well	700	14.9	23	21.9	13	14.9	14	18.7	13	22.0	0.18
G2, moderately	3379	71.9	66	62.9	59	67.8	53	70.7	35	59.3	
G3, poorly	621	13.2	16	15.2	15	17.2	8	10.7	11	18.6	
T stage											
T1	1795	35.0	61	52.1	34	35.1	33	40.7	26	38.8	0.03

© 2026 Yu S et al. *JAMA Network Open*.

T2	1169	22.8	17	14.5	27	27.8	16	19.8	13	19.4	
T3	1861	36.3	29	24.8	32	33.0	26	32.1	23	34.3	
T4	300	5.9	10	8.5	4	4.1	6	7.4	5	7.5	
N stage											
N0	2409	46.9	76	64.4	54	55.7	48	59.3	31	47.0	0.01
N1	2200	42.8	36	30.5	32	33.0	28	34.6	31	47.0	
N2	381	7.4	4	3.4	9	9.3	3	3.7	3	4.5	
N3	145	2.8	2	1.7	2	2.1	2	2.5	1	1.5	
M stage											
M0	4617	89.0	109	92.4	84	84.0	77	95.1	60	87.0	0.12
M1	572	11.0	9	7.6	16	16.0	4	4.9	9	13.0	
Clinical stage											
stage I	1610	31.0	57	48.3	30	30.0	33	40.7	23	33.3	0.001
stage II	1567	30.2	25	21.2	38	38.0	23	28.4	14	20.3	
stage III	1440	27.8	27	22.9	16	16.0	21	25.9	23	33.3	
stage IV	572	11.0	9	7.6	16	16.0	4	4.9	9	13.0	
Tumor location											
Upper	851	16.8	18	15.3	25	25.8	18	22.2	14	20.3	0.30
Middle	1927	37.9	54	45.8	39	40.2	32	39.5	26	37.7	
Lower	2252	44.3	45	38.1	33	34.0	30	37.0	28	40.6	
EG junction	49	1.0	1	0.9	0	0.0	1	1.2	1	1.5	
Treatment Method											
Operation	1671	32.2	24	20.3	29	29.0	13	16.0	11	15.9	<.001
Endoscopic resection	254	4.9	28	23.7	1	1.0	17	21.0	8	11.6	
Adjuvant	574	11.1	5	4.2	5	5.0	10	12.3	6	8.7	
Neoadjuvant	655	12.6	6	5.1	2	2.0	5	6.2	4	5.8	
CCRT	1290	24.9	31	26.3	50	50.0	27	33.3	28	40.6	
Others	117	2.3	6	5.1	2	2.0	3	3.7	0	0.0	

© 2026 Yu S et al. *JAMA Network Open*.

Best supportive care	628	12.1	18	15.3	11	11.0	6	7.4	12	17.4

CCRT, concurrent chemoradiation therapy; HN, Head & Neck; SD, standard deviation

© 2026 Yu S et al. *JAMA Network Open*.

eTable 5. Impact of prior stomach cancer on the prognosis of subsequent primary esophageal squamous cell carcinoma

	Overall survival				Esophageal cancer-specific survival			
Characteristics	Multivariate HR	lower 95% CI	upper 95% CI	p-value	Multivariate Cause-specific HR	lower 95% CI	upper 95% CI	p-value
Prior malignancy								
No	ref				ref			
Yes	1.15	0.87	1.51	0.33	2.63	1.27	5.44	0.009
Age (mean, SD)	1.03	1.02	1.03	<.001	1.03	1.01	1.05	0.01
Sex								
Male	0.99	0.82	1.20	0.95	0.91	0.44	1.88	0.79
Female	ref				ref			
Smoking								
Current smoker	1.22	1.08	1.37	0.002	1.27	0.75	2.14	0.37
Non-smoker	ref				ref			
Ex-smoker	1.00	0.88	1.12	0.94	0.78	0.48	1.28	0.32
Cormobidity								
Liver cirrhosis	1.51	1.23	1.87	<.001	2.35	1.06	5.17	0.03
Cerebrovascular disease	1.30	1.04	1.62	0.02	0.79	0.32	1.96	0.61
Grade (differentiation)								
G1, well	ref				ref			
G2, moderately	1.05	0.93	1.20	0.42	1.05	0.59	1.86	0.88
G3, poorly	0.96	0.82	1.13	0.65	1.06	0.54	2.07	0.87
Clinical stage								
stage I	ref				ref			
stage II	2.61	2.27	2.99	<.001	2.40	1.22	4.71	0.01

© 2026 Yu S et al. *JAMA Network Open*.

stage III	4.63	4.05	5.29	<.001	9.43	5.21	17.04	<.001
stage IV	8.53	7.29	9.99	<.001	31.38	16.57	59.42	<.001
Tumor location								
Upper	ref				ref			
Middle	1.03	0.91	1.16	0.64	2.11	1.24	3.59	0.006
Lower	0.92	0.81	1.04	0.17	1.13	0.65	1.97	0.67
EG junction	0.80	0.51	1.25	0.33	1.10	0.14	8.36	0.93

CI, confidence interval; HR, hazard ratio; SD, standard deviation

© 2026 Yu S et al. *JAMA Network Open*.

eTable 6. Impact of prior lung cancer and head & neck cancer on the prognosis of subsequent primary esophageal squamous cell carcinoma

	Overall survival				Esophageal cancer specific survival			
Characteristics	Multivariate HR	lower 95% CI	upper 95% CI	p value	Multivariate Cause-specific HR	lower 95% CI	upper 95% CI	p value
Prior malignancy								
No	ref				ref			
Yes	1.63	1.24	2.15	0.001	1.61	0.39	6.60	0.51
Age (mean, SD)	1.03	1.02	1.03	<.001	1.03	1.00	1.05	0.02
Sex								
Male	1.01	0.83	1.22	0.96	0.81	0.40	1.63	0.55
Female	ref				ref			
Smoking								
Current smoker	1.20	1.06	1.35	0.004	1.30	0.76	2.21	0.34
Non-smoker	ref				ref			
Ex-smoker	0.97	0.86	1.09	0.56	0.76	0.46	1.27	0.30
Cormobidity								
Liver cirrhosis	1.52	1.23	1.87	<.001	2.41	1.09	5.32	0.03
Cerebrovascular disease	1.30	1.05	1.62	0.02	0.85	0.34	2.13	0.74
Grade (differentiation)								
G1, well	ref				ref			
G2, moderately	1.07	0.94	1.21	0.34	1.16	0.63	2.14	0.64
G3, poorly	0.97	0.82	1.13	0.67	1.17	0.58	2.38	0.66
Clinical stage								
stage I	ref				ref			
stage II	2.58	2.25	2.96	<.001	2.14	1.08	4.23	0.03
stage III	4.61	4.03	5.27	<.001	8.38	4.61	15.24	<.001

© 2026 Yu S et al. *JAMA Network Open*.

stage IV	8.36	7.14	9.78	<.001	28.76	15.14	54.63	<.001
Tumor location								
Upper	ref				ref			
Middle	1.02	0.90	1.15	0.75	1.82	1.09	3.05	0.02
Lower	0.91	0.81	1.03	0.13	0.96	0.56	1.66	0.89
EG junction	0.77	0.49	1.23	0.28	0.95	0.13	7.18	0.96

CI, confidence interval; HR, hazard ratio; SD, standard deviation

© 2026 Yu S et al. *JAMA Network Open*.
