# Supplementary material for: Prior Cancer and Survival in Patients With Esophageal Squamous Cell Carcinoma
Source: JAMA Netw Open. 2026 Feb 20;9(2):e2560193. doi: 10.1001/jamanetworkopen.2025.60193 (PMC12924099; doi:10.1001/jamanetworkopen.2025.60193)
Supplement: Supplement 2. — Data Sharing Statement [file jamanetwopen-e2560193-s002.pdf]

Data Sharing Statement

Yu. Prior Cancer and Survival in Patients With Esophageal Squamous Cell Carcinoma. *JAMA Netw Open*. Published February 20, 2026. doi:10.1001/jamanetworkopen.2025.60193

Data
Data available: No

Additional Information
Explanation for why data not available: Data will be available upon reasonable request to the corresponding author.
